# Candelilla Wax and Glycerol Monostearate-Based Oleogels as Animal Fat Substitutes in Bologna Sausages

**DOI:** 10.3390/gels10060399

**Published:** 2024-06-13

**Authors:** Anda Elena Tanislav, Anca Alexandra Cornea, Eugen Dan Radu, Dorin Țibulcă, Vlad Mureșan, Elena Mudura

**Affiliations:** Food Engineering Department, Faculty of Food Science and Technology, University of Agricultural Sciences and Veterinary Medicine Cluj-Napoca, 3-5 Calea Mănăștur Street, 400372 Cluj-Napoca, Romania; anda.tanislav@usamvcluj.ro (A.E.T.); anca-alexandra.cornea@student.usamvcluj.ro (A.A.C.); eugen-dan.radu@student.usamvcluj.ro (E.D.R.); dorin.tibulca@usamvcluj.ro (D.Ț.); elena.mudura@usamvcluj.ro (E.M.)

**Keywords:** oleogel, fat substitution, meat composition, candelilla wax, glycerol monostearate, hardness, sunflower oil

## Abstract

The aim of this study was to produce Bologna sausages rich in unsaturated fatty acids and to evaluate this replacement on the structural characteristics. For the purpose of a comparative analysis, three different types of sausages were produced, distinct only in the type of fat used: I. sausages obtained with pork backfat (PBF), II. sausages produced with oleogel formed from refined sunflower oil and glycerol monostearate (GM_OG), and III. with candelilla wax oleogel (CW_OG). The meat composition was also analyzed to better understand the process in the dynamics and the finished products were analyzed both uncooked and cooked. The enhanced oil-binding capacity of oleogels suggests their potential value as substitutes for saturated fats (>99%). In terms of meat composition textural analysis, the highest hardness value was registered for PBF_C of 25.23 N, followed by a CW_OG_C of 13.08 N and a GM_OG_C of 12.27 N. However, adhesiveness, cohesiveness, springiness index, and gumminess showed similar values between samples. Reformulation of products with oleogels as a fat source abundant in mono- and polyunsaturated fatty acids resulted in uncooked products exhibiting reduced hardness values of 49.01 N (CW_OG_US) and 40.51 N (GM_OG_US), compared to 65.03 N (PBF_US). Color results of the cross-section color can indicate the potential for consumer acceptance due to the reduced color differences between the conventional and oleogel samples.

## 1. Introduction

The meat industry plays a crucial role in processing and providing nutrient-rich foods. Meat and its derived products serve as significant sources of essential macronutrients such as proteins and lipids, as well as vital micronutrients including amino acids, vitamins (particularly from the B group), and minerals (such as iron, selenium, and zinc). These nutrients are necessary for maintaining a proper human metabolism [1,2]. On the other hand, processed meat products frequently contain additives such as sodium chloride, nitrites, and phosphates, incorporated for diverse technological purposes [3]. Moreover, the higher level of fat content observed in processed meat products (reaching levels of up to 30% in emulsified meat products such as Bologna sausages) [4,5], predominantly comprising saturated fatty acids, substantially increase the susceptibility of developing various medical conditions such as cardiovascular diseases, type 2 diabetes, and obesity [6].

In order to limit the intake of saturated fatty acids, various organizations, including the World Health Organization, Food and Agricultural Organization [7], U.S. Department of Health and Human Services, and U.S. Department of Agriculture [8], issued some proposals. The aforementioned recommendations suggest a reduction in overall fat consumption to less than 30–35% of total caloric intake, limiting saturated fatty acids to less than 10%, and increasing the proportion of polyunsaturated fatty acids.

The substitution of animal fat in meat products presents a significant challenge for both the industry and scientific community. This is primarily due to the integral role that animal fat plays in maintaining the sensory attributes of the final product, an aspect highly valued by consumers. Consequently, any modifications in fat composition must be carefully considered to keep product quality and consumer satisfaction. Saturated fat, specifically pork backfat or beef fat, is an essential ingredient in emulsified meat products, as it significantly influences the texture, mouthfeel, juiciness, and savor [9]. Over the last several decades, various research has explored alternative replacements for saturated fatty acids in meat products, although with limited success. Water binders, while explored, have been found to compromise the product juiciness, mouthfeel, and shelf life due to the high moisture content in the product [9]. On the other hand, the addition of unsaturated fats (vegetable or fish oil) in liquid form presented sensory challenges, including increased hardness, reduced stability, color changes, and increased susceptibility to oxidation during storage [4,5].

Although it is challenging to find a suitable method for replacing saturated animal fats in meat products, there is a growing interest in studying a specific technology called oleogelation. This is evidenced by the reviews addressing the use of oleogels in food [10], the development of meat products by substituting saturated fatty acids with oleogels [11], the use of oleogels as innovative technologies to enhance the nutritional properties of meat products [12], and the examination of the structure, oxidative stability, and sensory characteristics of oleogels [13]. Oleogels represent semi-solid systems characterized by the immobilization of the lipid phase in a three-dimensional network [14]. These structured systems are versatile since they may provide a wide range of characteristics, depending on the type and amount of gelling agent and the characteristics of the oil used [5]. Oleogels have been successfully used to replace saturated fats in foods other than meat products, such as baked products [15], dairy products [16,17], breakfast spreads [18,19], and confectionery products [20,21]. In meat products, replacing saturated fats with oleogels presented the advantages of providing a textural structure and color that was comparable to the reference samples and showing a protective effect against lipid oxidation [5].

Regarding the use of oleogels to replace animal fat in Bologna-type sausages, Ferro et al. found that total substitution of saturated fat with glyceryl monostearate oleogel leads to a slight increase in hardness, but when just 50% of the fat was replaced, the texture of the sausages remained unchanged [5]. Also, consumer sensory analysis revealed a high level of acceptance. Comparable positive results were achieved by da Silva et al. regarding the sensory analysis when they substituted 50% of the pork backfat with an oleogel obtained from high oleic sunflower oil, water, and pork skin [22]. The use of oleogels obtained from conventional and high oleic soybean oil was studied by Tarté et al., who reported technological and sensory parameters similar to traditional Bologna sausages [23]. Regarding low-protein Bologna sausages, substitution of animal fat with soybean oil oleogels can result in improved stability of batter and a softer texture of the product. In contrast to the control sample, Bologna sausages produced using emulsion gels and reduced salt content exhibited considerable deterioration in sensory attributes, with flavor being the most significantly affected characteristic [2]. On the other hand, the emulsion gels obtained by Cîrstea Lazar et al., using olive, walnut, and chia oil, stabilized with chitosan, showed insignificant changes in terms of general acceptability, physico-chemical characteristics, and oxidation stability when pork backfat was completely substituted. The only notable differences were observed in terms of color, with a slight decrease in redness and an increase in both yellowness and lightness [4].

Aranda-Ledesma et al. conducted a study on the potential use of candelilla wax in the food sector. Their findings indicate that incorporating candelilla wax can lead to the production of safe and high-quality food products [24]. Candelilla wax (E 902) is recognized as a glazing agent in the European Union [25], but in the United States, it is GRAS-approved (generally recognized as safe) and is permitted to be used in a variety of foods without any restrictions other than those imposed by current good manufacturing practices [26]. The Food and Agriculture Organization (FAO) and the World Health Organization (WHO) also find it acceptable, although they do not provide a specific maximum daily intake [24]. Glycerol monostearate is a white powder that absorbs moisture, has a sweet taste, and is odorless. It is widely recognized as the most often used food emulsifier [27,28]. Distinct from its emulsifying properties, in the food industry, it may also serve as a gelling agent for oils [27]. The Panel of the European Food Safety Authority formed the conclusion that an acceptable daily intake dose for mono- and di-glycerides was unnecessary. Studies on short-term, toxicity, chronic effects, and reproductive effects have not shown any negative impacts [29]. Furthermore, the Food and Drug Administration states that the ingredient can be used in food products without any restrictions other than current good manufacturing practices [30].

The aim of this study was to totally substitute saturated fat with unsaturated fatty acids through the development and analysis of oleogels obtained from sunflower oil and glycerol monostearate/candelilla wax. The effectiveness of these oleogels was evaluated during the manufacturing process of Bologna sausages. According to the scientific literature, the research carried out on the replacement of animal fat was studied on different types of oleogels: pork skin, water, and high oleic sunflower oil [22]; soybean oil with rice bran wax [31,32]; monoglyceride with sunflower oil [5]; and pork skin, water, and amorphous cellulose [33]. In the present research, two types of oleogels were analyzed as animal fat replacers: I. with glycerol monostearate due to the favorable results reported [5] and II. with candelilla wax, because to our knowledge, oleogels obtained from sunflower oil and candelilla wax have not been reported as fat substitutes in Bologna sausages.

## 2. Results and Discussion

### 2.1. Oleogel Characterization

#### 2.1.1. Texture Profile Analysis

There were no significant differences between the values of the textural parameters for the two types of oleogels, obtained with different gelling agents. Hardness (the maximum load value of the first compression cycle) and adhesiveness (area under the load vs. distance curve) [34] showed higher values for candelilla wax oleogel (CW_OG)—28.96 N and 40.87 mJ, respectively, compared to glycerol monostearate oleogel (GM_OG) values of 12.27 N and 34.80 mJ (Table 1). Lim et al., reported similar values of hardness and adhesiveness for candelilla wax oleogel structured with canola oil—25.12 N and 0.14 N·s [35]. Chia seed oil and glyceryl monostearate oleogel showed lower hardness values (5.15–7.32 N depending on the cooling rate), but similar cohesiveness values (0.17–0.25) with our study [36]. A harder oleogel could retain its properties on the technological process and could be more suitable for the specific aspect of animal fat in meat products. The higher adhesiveness values may be explained by the increased proportion of the structuring agent (10%), as indicated by Pandolsook’s and Kupongsak’s findings, where adhesiveness increased when gelling agent concentration increased from 3% to 9% [37]. On the other hand, the cohesiveness (hardness work done cycle 2/hardness work done cycle 1) [34] of the oleogels registered a slightly higher value when the oil was structured with 10% glycerol monostearate—0.17 as opposed to the structuring with candelilla wax—0.12. Lim et al. obtained comparable results for the cohesiveness value when canola oil was structured with carnauba wax (0.20) and beeswax (0.33) [35], respectively.

#### 2.1.2. Oil Binding Capacity

Oil binding capacity (OBC) expresses the strength and nature of oil–gelators interactions [38]. Because oleogels comprise a significant volume of liquid (90% in the current study), a high OBC is important. A low oil binding capacity may result in oil leaking into the food matrix to which oleogel is added, an undesirable consequence which tries to be reduced when using matrices containing substantial quantities of oil. For the formulated oleogels, the oil binding capacity was not significantly different between samples, with a value of 100% for CW_OG and 99.28% for GM_OG, indicating the potential of application as saturated fat substitutes (Table 1). According to Su et al., the relationship between hardness and OBC is tight, so higher hardness indicates a more compact network structure of the gels [39]. In our study, the oleogel with candelilla wax presented a higher hardness, and consequently, a more compact network. Similarly, oleogels with linseed and canola oil structured with beeswax and shellac wax [38] or sunflower oil oleogels (carnauba wax/β-sitosterol: beeswax/β-sitosterol: lecithin) presented a high oil binding capacity (OBC > 96.85%) [40], and oleogels with flaxseed oil, candelilla wax, and flaxseed gum also presented a high OBC, >80% [39]. Keskin and Yilmaz prepared sunflower oil oleogels with glycerol monostearate and different amphiphiles and reported higher oil binding capacities, similar to our study, of 99.3% and 100% [27].

### 2.2. Meat Composition Characterization

#### 2.2.1. Texture Profile Analysis

The effects of pork backfat replacement with glycerol monostearate or candelilla wax oleogel on the textural properties of Bologna sausages composition are presented in Table 2. Adhesiveness, cohesiveness, and springiness index were not significantly affected by the addition of oleogels. All textural properties decreased when animal fat was 100% replaced with oleogels, except cohesiveness. Ferro et al., reported less cohesive samples by 100% animal fat substitution, while springiness was minimally influenced [5].

The highest hardness value was recorded for the sample with pork backfat—25.23 N (PBF_C). The meat compositions with oleogels presented similar values of 12.27 N for glycerol monostearate composition (GM_OG_C) and 13.08 N for candelilla wax composition (CW_OG_C). On the other hand, Barbut and Marangoni reported higher hardness values when beef fat was converted to organogel, but similar to our study, the comminuted meat systems obtained with organogels prepared from canola oil were softer. This may be due to larger fat globules, which lead to less firm meat products [41]. Also, the degree of saturation of the fat/oil has a significant role in determining the texture of the finished products [42]. The adhesiveness values were similar for all three compositions, with a maximum value of 72.27 mJ for CW_OG_C, followed by 53.33 mJ for PBF_C and 43.90 mJ for GM_OG_C.

The springiness index (springiness/distance at target) [34] values were not influenced by the type of fat used. Springiness index slightly decreased when pork backfat was replaced with oleogel from 1.00 to 0.99 (CW_OG_C) and 0.91 (GM_OG_C), so the meat compositions had the same elasticity, regardless of the type of fat source. Similar to hardness, gumminess (hardness*cohesiveness) [34] showed the highest value for the sample with pork back fat, followed by CW_OG_C and GM_OG_C.

#### 2.2.2. Fat Loss

The effects of 100% animal fat replacement resulted in an increase in the fat loss of meat compositions. This suggests distinctive interactions between the protein matrix and oleogels, opposed to interaction between protein and animal fat. The highest fat loss was recorded for the sample with oleogel with glycerol monostearate—7.75%, followed by the meat composition with oleogel with candelilla wax—5.81%. These values are significantly different from the fat loss recorded for the conventional composition obtained with pork backfat—1.05%, but they still remained below 8%, indicating the potential of using oleogels in the formulation of healthier meat products. On the other hand, Tarté et al. reported an improved batter stability when animal fat was replaced by oleogels (soybean oil and rice bran wax) [23] and in the study conducted by Ferro et al., the pork fat replacement resulted in an increased emulsion stability [5]. These differences can be attributed to the type of meat used and their fat content (and whether the meat was defatted).

#### 2.2.3. Rheology

Amplitude sweep tests were the first step in rheological analysis to determine the linear viscoelastic region (LVR) for each sample. Oscillatory strains ranging from 0.01% to 100% were applied and the resulting values of the storage (G′) and loss (G″) modulus have been determined. The LVR is identified as a plateau at which G′ and G″ remain constant prior to the deformation that causes both moduli to decrease [27]. The LVR indicates the range in which the sample could be stressed without destroying its structure. The oscillatory strains’ LVR was 0.01% for oleogels meat compositions and 0.1% for pork backfat meat composition. Thus, frequency tests were performed at a constant LVR of 0.01% to include all samples.

**Figure 1:**
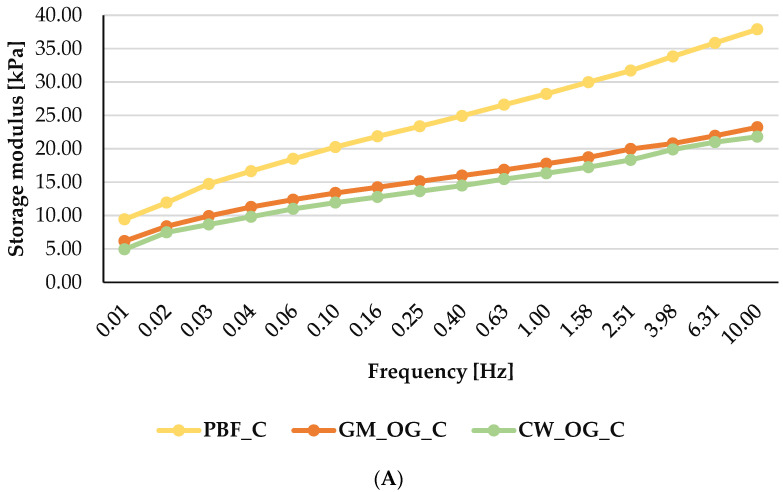

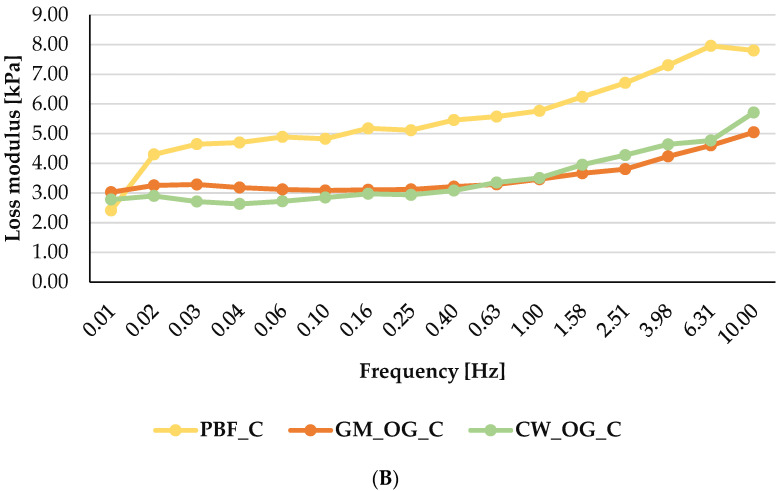
Storage modulus G′ (**A**) and loss modulus G″ (**B**) curves for meat compositions during the frequency test. PBF_C—meat composition with pork backfat; GM_OG_C—meat composition with glycerol monostearate oleogel; CW_OG_C—meat composition with candelilla wax oleogel.

To complete the frequency tests, oscillatory strains according to the LVR and in the range of 0.01–10 Hz were applied for each sausage composition at a temperature of 4 °C. The structure of meat batter is mainly determined by the quantity and quality of fat and meat [43]. To better understand the textural properties, the viscoelastic properties were performed by measuring the frequency sweep of the meat compositions. Figure 1A,B shows the storage modulus (G′) and the loss modulus (G″) for the sausage compositions. All formulations presented mechanical characteristics of gels, with G′ values higher than G″, the moduli being almost parallel to each other. The storage modulus (G′) indicates the elastic components and implies the properties of a sample in the solid state, while the loss modulus (G″) suggests the viscous component and explains the properties of a sample in the liquid state [27]. Therefore, G′ > G″ values indicate that meat compositions belong to viscoelastic solids. Regardless the frequency range, no crossover point (G′ = G″) was observed, suggesting that each sample maintained its structured state. The storage modulus values increased slightly and gradually with the frequency range used in the analysis (0.01–10 Hz), while the loss modulus showed slight oscillations. These rheological properties serve as an indicator of the degree of intertwining among protein molecules at the interface between oil and water. They are also associated with the development of the emulsion structural network [5]. G′ values presented the following trend: PBF_C > GM_OG_C > CW_OG_C, while for G″, PBF_C presented the highest values, and GM_OG_C presented higher values up to the frequency of 1 Hz, where it crossed with CW_OG_C, whose values dominated up to 10 Hz. The results were in accordance with the hardness values, where the sample with animal fat presented the highest value, and the samples with oleogels presented lower, but similar, statistically insignificant values. Similarly, de Souza Paglarini et al. reported the highest values for G′ and G″ for the pork fat sample, in contrast to those reformulated with gel emulsions [43]. In the study carried out by Ferro et al., they reported the highest values for G′ and G″ for the sample with oleogel, followed by the mixture of pork fat and oleogel (1:1) and only then, when pork backfat was used alone, as a fat source [5].

#### 2.2.4. Color

For the meat composition, the instrumental L* color values showed that PBF_C was significantly lighter (the whiteness also showed the higher value—62.40) and GM_OG_C and CW_OG_C were darker (Table 2). Barbut and Marangoni reported that the conversion of vegetable oils to organogels significantly reduced the lightness values, probably due to larger fat globules causing less light reflection compared to the more numerous smaller fat globules (oil) [41]. Redness (a* values) is mainly related to the amount of red blood pigment (myoglobin) in the meat, and was not significantly affected by replacement with glycerol monostearate oleogel (GM_OG), when it produced a higher value—6.32, compared to 5.98 for PBF. Yellowness (b* values) was lower for meat compositions with oleogel, compared to PBF_C (10.25). A similar trend was observed for a* values in Barbut’s and Marangoni’s study, but for the b* values, they reported lower values for comminuted meat system with beef fat [41]. Chroma values (C*) represent the color intensity, with the highest value for the meat composition with candelilla wax oleogel (13.61), followed by similar values between the samples with glycerol monostearate oleogel (7.78) and pork backfat (7.56). Regarding the color, differences visible to the human eye were perceived between the samples because the calculated ΔE parameters were 11.99 for GM_OG_C and 12.34 for CW_OG_C.

### 2.3. Finished Product Characterization

#### 2.3.1. Texture Analysis

**Figure 2:**
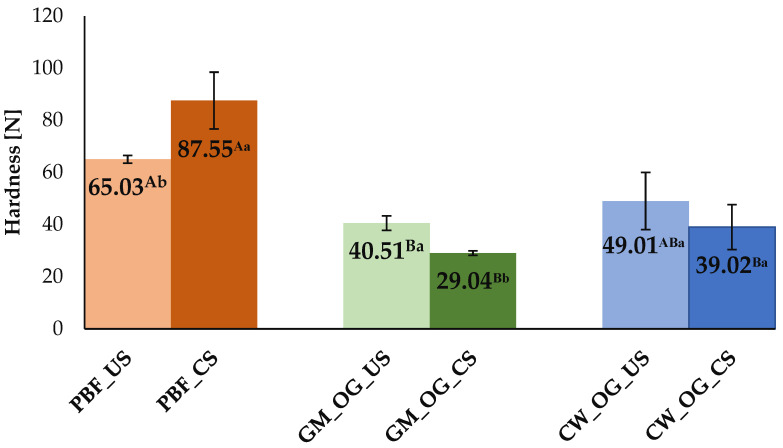
Hardness for uncooked and cooked Bologna sausages. Values are expressed as mean ± standard deviation. PBF_US—uncooked sausages with pork backfat; GM_OG_US—uncooked sausages with glycerol monostearate oleogel; CW_OG_US—uncooked sausages with candelilla wax oleogel; PBF_CS—cooked sausages with pork backfat; GM_OG_CS—cooked sausages with glycerol monostearate oleo-gel; CW_OG_CS—cooked sausages with candelilla wax oleogel. For each characteristic, identically superscript capital letters indicate no significant differences (*p* > 0.05) between samples (PBF_US vs. GM_OG_US vs. CW_OG_US; PBF_CS vs. _GM_OG_CS vs. CW_OG_CS). Identically superscript lowercase letters indicate no significant differences (p >0.05) between uncooked and cooked samples (PBF_US vs. PBF_CS; GM_OG_US vs. GM_OG_CS; CW_OG_US vs. CW_OG_CS).

Saturated fatty acids have a major impact on the textural properties of meat products, since animal fat significantly influences the texture of these products. Thus, substituting these fats with unsaturated fatty acids may result in products that are less firm and stable, which is a disadvantage from a technological point of view despite their nutritional value [44]. Textural characteristics constitute the most significant challenges in food products reformulation. Hardness results for uncooked and cooked Bologna sausages are presented in Figure 2. The use of oleogels as fat source rich in mono- and polyunsaturated fatty acids led to products with lower hardness values in uncooked products, from 65.03 N for pork backfat uncooked sausages (PBF_US) to 49.01 N for candelilla wax uncooked sausages (CW_OG_US) and 40.51 N for glycerol monostearate uncooked sausages (GM_OG_US). The cooking process for consumption led to an increase in hardness for the sample obtained with animal fat to 87.55 N (PBF_CS), while for the reformulated samples with gelled systems, it decreased to 39.02 N (CW_OG_CS) and 29.04 N (GM_OG_CS), respectively. The differences were followed between all uncooked and cooked samples (PBF_US/CS vs. GM_OG_US/CS vs. CW_OG_US/CS), as well as in relation to the cooking treatment for each sample (PBF_US vs. PBF_CS, GM_OG_US vs. CW_OG_CS, GM_OG_US vs. CW_OG_US). There were no significant differences in hardness identified when comparing the conventional uncooked sample and the sample with oleogel with candelilla wax and between CW_OG_US and CW_OG_CS. Similar to our study, the replacement of animal fat with an emulsion gel in Bologna sausages decreased hardness [1]. No differences in hardness were reported for chicken-based Bologna sausages when pork backfat was replaced with oleogel (soybean oil and rice bran wax) [23] and in frankfurters when animal fat was replaced with sunflower oil oleogels [45]. On the other hand, higher hardness values were reported when pork fat was replaced with glycerol monostearate and sunflower oil oleogel [5], with an emulsion obtained with pork skin, sunflower oil, and water [22], and with emulsion gels [4] in Bologna sausages. Wolfer et al. reported higher hardness compared to the control (with pork backfat) in frankfurters when a replacement was made for oleogel with 10% rice bran wax and soybean oil, but less hard when the oleogel was formed with 2.5% rice bran wax [46]. In the study conducted by Ferro et al., according to the sensory analysis, the use of glyceryl monostearate-based oleogels made the samples easier to slice, this being important in terms of consumer acceptance [5].

The contradictory results may be due to the different composition of the oleogels (including the type of oil and the structuring agent), the differences that appear in the production technology, the percentage of fat replaced, and the type of meat used (including the fat contained in certain types of meat). In the present study, since the only difference between the samples was the type of fat used, the results obtained can be associated with the properties of the oleogel, the animal fat, and their interaction with the protein matrix of the meat composition, respectively. Considering that by using liquid oil, harder products are obtained due to the smaller globules of fats formed, oleogels have the advantages of forming a larger surface area available for the interactions between fats and proteins, which can lead to a higher resistance to compression [5]. Nevertheless, the variations in hardness were relatively minor and probably would not have been noticed by the majority of consumers.

#### 2.3.2. Color

Color is an essential parameter when food products are reformulated because it influences the consumer acceptability and desire to buy the product. The results of color parameters (L*, a*, b*, ΔE, Whiteness, C*) was influenced by oleogel addition (Table 3). The external surface color varied more than the cross-section color. In general, replacing pork backfat with sunflower oil oleogels resulted in lighter (higher L* values), less red and more yellow Bologna sausages. Similarly, Wolfer et al., and da Silva et al., reported higher darkness and redness in pork backfat frankfurters than in soybean oil, and rice bran wax oleogel treatments, respectively in Bologna sausages with 100% substitution of pork backfat with oleogel (pork skin, water, and high oleic sunflower oil) [22,46]. In the present study, the use of sunflower oil in the form of oleogels led to lighter sausages, but with insignificantly different values from the sample with pork backfat. Among all treatments, for the external surface color, GM_OG_US was the lightest (57.74) and for the cross-section color CW_OG_CS (60.82). These small differences may not be observed if replicating the experiment using liquid oils from distinct production batches and/or suppliers [23]. Whiteness values increased for samples with oleogels showing that reformulated sausages were lighter (wither color) than the conventional sample. These results are in agreement with the results of the study carried out by Ferro et al., [5]. For the a* values in the cross-section of the sausages, the values decreased with the replacement of animal fat, but not statistically significant. However, for the external surface, the values were significantly different from the conventional sample for both uncooked and cooked samples (13.59 for PBF_US, 9.72 for GM_OG_US, 10.05 for CW_OG_US; 17.64 for PBF_CS, 12.87 for GM_OG_CS, 13.60 for CW_OG_CS). A similar decrease in redness was noticed in oleogel frankfurters [45,47,48] and Bologna sausages [49]. The parameter b* showed higher values for PBF_US and PBF_CS for external surface color, but for the cross-section color, the values increased with the replacement of animal fat, from 6.16 to 7.77 (GM_OG_US) and 8.20 (CW_OG_US) and from 8.53 to 11.65 (GM_OG_CS) and 9.76 (CW_OG_CS). An increase in the b* values at partial or total replacement of pork backfat with oleogel was reported by da Silva et al., and de Oliveira Faria et al., in Bologna type sausages [22,33]. The lightness increase of the products can be attributed to the glassy appearance of the oleogels and the increase in the b* parameter (yellow) to the yellow-cream color specific to oleogels and sunflower oil [50]. Variations in luminosity can also be explained by the size of the fat globules, because smaller fat globules correspond to increased light reflectance [44].

The ∆E values indicate the color differences between the conventional sample and the samples reformulated with oleogels. Values of 2 to 3.5 indicate that an inexperienced observer is able to identify the color difference, 3.5 to 5 indicate that any observer can quickly perceive the difference, and values higher than 5 indicate that the human eye can distinguish between two different colors [5]. ∆E results for external surface color indicated that the total replacement of animal fat can be perceived by consumers, the most different samples comparing to PBF_US/CS being GM_OG_CS (∆E = 7.76). However, Bologna sausages obtained with oleogel with candelilla wax presented a color closer to the conventional sample. Regarding the cross-section color of the sausages, ∆E values were between 2 and 3.5 with the exception of the sample with oleogel with candelilla wax in uncooked sausages (∆E = 4.81). Similar ∆E values were obtained for Bologna sausages in which emulsion gels was used as pork backfat replacer [43].

In the reformulated sausages, the replacement of pork backfat with oleogels did not always provide a similar color parameter because the color effect of reformulated sausages containing pork back fat was not completely replicated by oleogels.

## 3. Conclusions

This study enhances the knowledge regarding the reformulation of meat products incorporating alternative lipid sources. The research demonstrated the potential use of oleogels in meat products with reduced saturated fat content. Oleogels obtained with sunflower oil and glycerol monostearate or candelilla wax can be used as potential substitutes for animal fat in Bologna sausages. The use of sunflower oil, due to the content of mono and polyunsaturated fatty acids, can improve the lipid profile of the finished products. The results showed that the oleogels were stable and well structured, with an oil loss below 0.8%, thus indicating the potential for application as fat substitutes. Through the lipid reformulation, although the hardness of the meat compositions decreased, did not significantly change their adhesiveness, cohesiveness, and springiness index. The obtained meat compositions, although they showed higher fat loss in contrast to the conventional sample, can be considered stable since the loss was below 8%. The total substitution of pork fat with candelilla wax oleogel was possible without significantly affecting the hardness of uncooked sausages. In general, the brightness and yellow color increased, while the redness decreased slightly for the reformulated products, and the color difference in the section was not strongly affected by the replacement. Candelilla wax-based oleogels can be considered more suitable to develop meat products with a low saturated fat content, with a higher potential to maintain the characteristics of the conventional product. Due to the high amount of unsaturated fatty acids, which are more prone to oxidation, future studies targeting oxidation in meat preparations would be necessary.

## 4. Materials and Methods

### 4.1. Materials

The oleogelators used were candelilla wax from Kahlwax GmbH & Co. KG. (Trittau, Germany), and purified glycerol monostearate from ThermoFisher GmbH (Waltham, MA USA). Pork leg (boneless and fatless) and pork backfat were purchased from a local butcher store (Cina Carmangerie, Cluj-Napoca, Romania). The refined sunflower oil (92 g fat/100 mL oil) used to obtain oleogels and the auxiliary materials for Bologna sausages production (sugar, nutmeg, allspice, red sweet pepper powder, garlic powder, red wine) were purchased from a local store. The polyphosphate was procured from Solina (Solina Romania—Phoenix Factory—Alba Iulia, Romania). Bologna sausages were manufactured in the Meat Pilot Station of the Faculty of Food Science and Technology, University of Agricultural Sciences and Veterinary Medicine of Cluj Napoca, Romania.

### 4.2. Preparation of Oleogels

Oleogels were prepared using 10% candelilla wax or glycerol monostearate and refined sunflower oil. The gelling agents and their concentration were chosen based on previous consultation with the literature and above the critical gelation point [24,51]. The oleogelation process was carried out with a magnetic stirrer (IKA^®^ C-MAG HS7, IKA-Werke GmbH & Co. KG, Staufen, Germany) at 500 rpm and a heating temperature of 80 °C, until the oleogelators was completely melt and dissolute. The oleogels were cooled at 4 °C for 24 h, and then analyzed or used to prepared the sausages. The samples were coded as: oleogel with candelilla wax (CW_OG) and oleogel with glycerol monostearate (GM_OG).

### 4.3. The Manufacturing Process of Bologna Sausages

**Figure 3:**
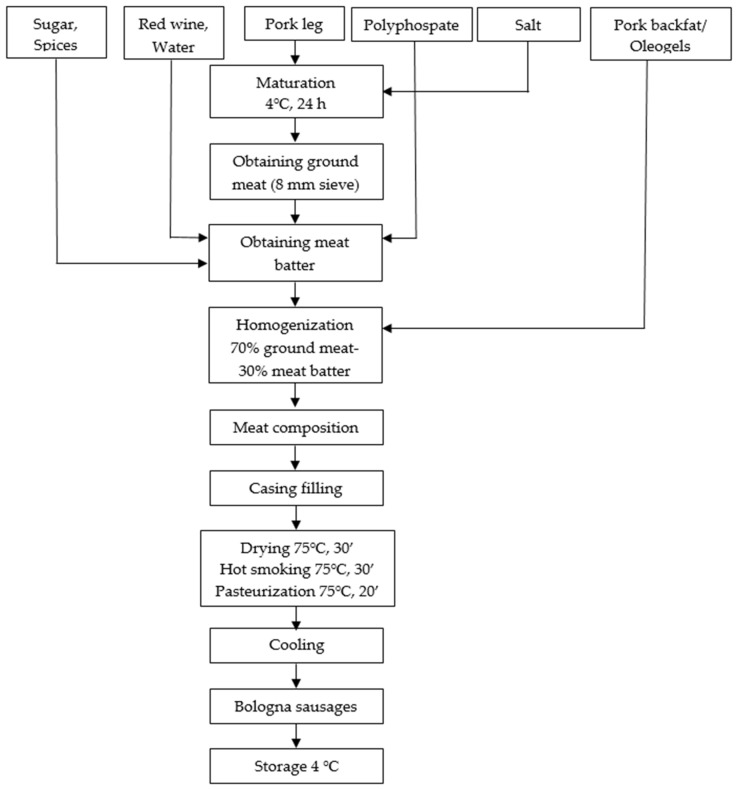
Process flow diagram of Bologna sausages.

Bologna sausages were manufactured according to the process flow diagram (Figure 3) and the ingredients of the formulations are presented in Table 4. Boneless and fatless pork leg was thawed at 18–20 °C for 24 h before being cut into pieces and combined with a 2% salt solution and matured for 24 h at 4 °C. The final composition consists of 70% ground meat and 30% meat batter. In order to obtain ground meat from the salted and matured meat, a meat grinder with an 8 mm sieve was used (Bizerba SE & Co. KG, Balingen, Germany). To prepare the meat batter, ground meat, spices, polyphosphate, water, and wine were added to the bowl cutter (Meprotec GmbH, Pasching, Austria) and finely chopped until a paste with adhesive properties, and homogenous structure was obtained. The oleogels or pork backfat (30%) were added after the meat batter was obtained, during the homogenization operation. After the homogenization process, samples were collected from each sausage composition in order to be analyzed before the applied heat treatment. The composition was stuffed in natural casings (pork intestines with 20 mm diameter) which were then tied (200 mm length) and if air bubbles were observed under the casing, the respective portions were punctured. The sausages were placed on a meat rack and subjected to a series of thermal treatments in a smoking and scalding chamber (H. Maurer & Söhne Rauch- und Wärmetechnik GmbH & Co. KG, Reichenau, Germany): air drying at 75 °C for 30 min, hot smoking at 75 °C for 30 min and pasteurization in a water bath at 75 °C for 20 min. The products were cooled, vacuumed in special bags, and stored between at 2–4 °C. The tests carried out on the meat compositions were performed on the same day of manufacture, and finished products analysis 24 h after production. The manufacturing recipe was taken from (Erwin and Hermann, 1987).

### 4.4. Oleogel Characterization

#### 4.4.1. Texture Profile Analysis

A texture analyzer (Brookfield CT3, Brookfield Engineering Labs, Middleboro, MA, USA) was used to determine the textural characteristics of the oleogel samples according to the method described by Monto et al., with slight modifications [51]. The samples were kept overnight at 4 °C and tested from this temperature. The TA11/1000-cylinder probe (25.4 mm D, 35 mm L) was used, with the following measurement parameters: test speed of 1 mm/s, return speed of 1 mm/s, compression ratio of 70%, and trigger load of 5 g. Hardness, adhesiveness, and cohesiveness were the parameters followed in this analysis.

#### 4.4.2. Oil Binding Capacity

The oil binding capacity was measured by centrifugation based on the method provided by Li et al., [52]. Melted oleogel was added and weighted in an empty tube (m1) and allowed to settle under the same oleogelation conditions (4 °C for 24 h). The mass of the tube containing the gel was weighed and recorded as m2. The sample was put in a Hettich Universal 320R centrifuge (Andreas Hettich GmbH & Co. KG, Tuttlingen, Germany) and centrifuged at 9000 rpm for 15 min at 4 °C. After centrifugation, the tubes were inverted for 30 min to drain the liquid oil, then weighed, and the mass representing m3 was recorded. The oil retention rate was calculated using the following formula: OBC [%] =100 − (m2 − m3)/m1) × 100, where: m1 is the mass of the sample, m2 is the total mass of the centrifuge tube with oleogel and m3 is the total mass after draining the liquid oil.

### 4.5. Meat Composition Characterization

#### 4.5.1. Texture Profile Analysis

Thermally non-treated meat composition (the sample was taken in the meat pilot station after the homogenization process) was also analyzed, to study the changes that occur in the dynamics of the process. The determination was made by subjecting the meat compositions (40 mm height × 45 mm diameter) to a force that achieves a deformation of 95% from the sample surface, with the cylindrical probe TA11/1000 which is attached to a 10 kg compression cell [53]. Deformation (in two cycles) was performed at a speed of 1 mm/s. The texturometer (Brookfield CT3, Brookfield Engineering Labs, Middleboro, MA, USA) records the load [N] as a function of time [s]. The parameters monitored in this textural analysis were: hardness, adhesiveness, cohesiveness, springiness index, and gumminess. Samples were stored at 4 °C and analyzed directly from this temperature.

#### 4.5.2. Fat Loss

Fat loss was determined by centrifugation of Bologna sausages meat compositions. Approximately 15 g of each sample was placed in Falcon screw-cap conical tubes (50 mL), placed in the DLAB DM0412 centrifuge (DLAB Scientific Co., Ltd., Beijing, China) and subjected to a speed of 4500 rpm for 30 min according to the method described by Jiménez-Colmenero et al., with slight modifications [54]. After centrifugation, the released fat was drained by inverting the tubes for 30–35 min. Comparison of the sample mass before and after a centrifugation cycle allowed the determination of fat loss from the initial sample mass: Fat loss [%] = (A − B)/C*100, where: A = initial mass (sample + tube before centrifugation, in grams); B = final mass (sample + tube after centrifugation and drainage, in grams); C = sample mass (in grams).

#### 4.5.3. Rheology

Changes in the dynamic viscoelastic properties of conventional and oleogel meat compositions were determined using an Anton Paar MCR 302 (Anton Paar, Graz, Austria) rheometer and serrated plate-plate geometry (PP35/2). The samples were placed between the plates and compressed to 10 mm gap and the excess sample was trimmed. The amplitude sweep test was performed in the range 0.01–100% to determine the linear viscoelastic region at a frequency of 1 Hz. The frequency sweep tests were performed at a constant shear strain of 0.01% in the frequency range 0.01–10 Hz at 4 °C [5]. Storage modulus (G′) and loss modulus (G″) were recorded.

### 4.6. Finished Products Characterization

#### Texture

Textural analysis was performed on both uncooked and cooked Bologna sausages (boiling for 15 min) by compressing the prototypes (50 mm length × 30 mm diameter) with the TA7 acrylic blade [55]. The compression of the samples was carried out in a single cycle, with a speed of 1 mm/s. The parameter monitored in the textural analysis of sausages was hardness. After obtaining, the samples were stored in vacuumed bags at 2–4 °C, being analyzed directly from this temperature and for the cooked samples after boiling and cooling. For this determination, the casing was not removed.

### 4.7. Color

Color measurement of oleogels, meat compositions and finished products (external surface and cross-section, uncooked and cooked samples) was performed with the NR200 portable colorimeter (3NH, Shenzhen, China). Lightness L*, a* (−a greenness, +a redness) and b* (−b blueness, +b yellowness) were measured using the CIE (Commission Internationale de l’eclairage of France) color system. The instrument performs an automatic calibration (L* = 0, a* = 0 and b* = 0). L*, a*, b* values were provided by the instrument software.

The color difference (ΔE) between conventional sample and oleogel samples was calculated as ΔE = √ (L × R − L × E)^2^ + (a × R − a × E)^2^ + (b × R − b × E)^2^, where L × R, a × R, b × R represents the mean values of the animal fat sample and L*E, a*E, b*E represents the mean values of each oleogel samples [56].

Whiteness was calculated for each sample as Whiteness= 100 − √ (100 − L*)^2^ + (a*)^2^ + (b*)^2^, where L*, a*, b* values represent the mean values [5].

Chroma or saturation index (C*) was determined for each sample as C* = [(a*^2^ + b*^2^)^1/2^], where a* and b* values represent the mean values [23].

### 4.8. Statistical Analysis

Analysis was performed in triplicate except for color which was performed in six repetitions. Differences were analyzed using Minitab 19 Statistical software. One-way analysis of variance (ANOVA) and Tukey’s comparison test at a significance level of *p* < 0.05 were used. All results were presented as mean ± standard deviation.

## Figures and Tables

**Table 1:** Texture profile analysis and oil binding capacity of oleogels.

Parameters	GM_OG	CW_OG
Texture profile analysis		
Hardness [N]	12.27 ^A^ ± 0.42	28.96 ^A^ ± 0.33
Adhesiveness [mJ]	34.80 ^A^ ± 13.58	40.87 ^A^ ± 11.61
Cohesiveness [n.a]	0.17 ^A^ ± 0.04	0.12 ^A^ ± 0.04
Oil binding capacity [%]	99.28 ^A^ ± 0.44	100.00 ^A^ ± 0.00

Values are expressed as mean ± standard deviation. GM_OG—glycerol monostearate oleogel; CW_OG—candelilla wax oleogel. For each characteristic, identically superscript capital letters indicate no significant differences (*p* > 0.05) between samples.

**Table 2:** Textural analysis, emulsion stability, and color for thermally non-treated meat compositions.

Parameters	PBF_C	GM_OG_C	CW_OG_C
Texture profile analysis			
Hardness [N]	25.23 ^A^ ± 6.82	12.27 ^B^ ± 2.35	13.08 ^B^ ± 1.94
Adhesiveness [mJ]	53.33 ^A^ ± 24.43	43.90 ^A^ ± 14.64	72.27 ^A^ ± 10.78
Cohesiveness [n.a]	0.51 ^A^ ± 0.06	0.62 ^A^ ± 0.07	0.62 ^A^ ± 0.09
Springiness index	1.00 ^A^ ± 0.01	0.91 ^A^ ± 0.06	0.99 ^A^ ± 0.04
Gumminess [N]	12.75 ^A^ ± 3.00	7.62 ^B^ ± 1.29	8.00 ^AB^ ± 0.68
Fat loss [%]	1.05 ^C^ ± 0.05	7.75 ^A^ ± 0.74	5.81 ^B^ ± 0.32
Color			
L*	64.33 ^A^ ± 1.46	52.42 ^B^ ± 0.80	52.11 ^B^ ± 1.09
a*	5.98 ^A^ ± 0.38	6.32 ^A^ ± 0.37	5.33 ^B^ ± 0.41
b*	10.25 ^A^ ± 0.43	8.94 ^B^ ± 0.46	8.66 ^B^ ± 0.97
ΔE	-	11.99	12.34
Whiteness	62.40	51.17	51.04
C*	7.56	7.78	13.61

Values are expressed as mean ± standard deviation. PBF_C—meat composition with pork backfat; GM_OG_C—meat composition with glycerol monostearate oleogel; CW_OG_C—meat composition with candelilla wax oleogel. For each characteristic, identically superscript capital letters indicate no significant differences (*p* > 0.05) between samples. ΔE indicates color differences of the samples in comparison to PBF_C. C* represent the color intensity.

**Table 3:** External surface and cross-section color for uncooked and cooked Bologna sausages.

Parameters	PBF_US	GM_OG_US	CW_OG_US	PBF_CS	GM_OG_CS	CW_OG_CS
External surface color						
L*	52.62 ^Aa^ ± 4.88	57.74 ^Aa^ ± 4.08	57.57 ^Aa^ ± 1.03	49.53 ^Ba^ ± 1.83	55.55 ^Aa^ ± 2.24	53.16 ^Ab^ ± 1.87
a*	13.59 ^Ab^ ± 2.01	9.72 ^Bb^ ± 0.97	10.05 ^Bb^ ± 1.22	17.64 ^Aa^ ± 1.09	12.87 ^Ba^ ± 1.12	13.60 ^Ba^ ± 1.88
b*	14.79 ^Ab^ ± 1.39	12.51 ^Bb^ ± 0.89	15.12 ^Ab^ ± 0.99	18.16 ^Aa^ ± 0.61	17.08 ^Aa^ ± 0.91	17.50 ^Aa^ ± 1.18
ΔE	-	6.81	6.09	-	7.76	5.47
Whiteness	48.54	54.87	53.85	43.53	50.67	48.18
C*	17.56	12.23	13.82	7.01	10.22	15.37
Cross-section color						
L*	55.10 ^Aa^ ± 3.56	56.26 ^Aa^ ± 6.58	59.39 ^Aa^ ± 2.65	58.70 ^Aa^ ± 3.60	59.09 ^Aa^ ± 1.40	60.82 ^Aa^ ± 2.51
a*	12.29 ^Ab^ ± 0.89	12.80 ^Aa^ ± 2.13	11.51 ^Ab^ ± 0.89	15.18 ^Aa^ ± 1.29	14.28 ^Aa^ ± 1.12	14.12 ^Aa^ ± 1.15
b*	6.16 ^Bb^ ± 1.13	7.77 ^Ab^ ± 0.87	8.20 ^Aa^ ± 1.07	8.53 ^Ba^ ± 0.62	11.65 ^Aa^ ± 2.64	9.76 ^ABa^ ± 1.56
ΔE	-	2.05	4.81	-	3.27	2.67
Whiteness	53.04	53.76	57.00	55.18	55.13	57.23
C*	19.75	20.07	15.17	11.19	24.03	17.97

Values are expressed as mean ± standard deviation. PBF_US—uncooked sausages with pork backfat; GM_OG_US—uncooked sausages with glycerol monostearate oleogel; CW_OG_US—uncooked sausages with candelilla wax oleogel; PBF_CS—cooked sausages with pork backfat; GM_OG_CS—cooked sausages with glycerol monostearate oleogel; CW_OG_CS—cooked sausages with candelilla wax oleogel. For each characteristic, identically superscript capital letters indicate no significant differences (*p* > 0.05) between samples (PBF_US vs. GM_OG_US vs. CW_OG_US; PBF_CS vs. _GM_OG_CS vs. CW_OG_CS). ΔE indicates color differences of the samples in comparison to PBF_US. Identically superscript lowercase letters indicate no significant differences (*p* > 0.05) between uncooked and cooked samples (PBF_US vs. PBF_CS; GM_OG_US vs. GM_OG_CS; CW_OG_US vs. CW_OG_CS). ΔE indicates color differences of the samples in comparison to PBF_US/CS. C* represent the color intensity.

**Table 4:** The recipe used in the production of Bologna sausages using oleogels or pork backfat (expressed in kg).

Ingredients	PBF	GM_OG	CW_OG
Boneless and fatless pork leg	70	70	70
Pork backfat	30	-	-
Glycerol monostearate oleogel	-	30	-
Candelilla wax oleogel	-	-	30
Sugar	1.430	1.430	1.430
Nutmeg	0.118	0.118	0.118
Allspice	0.034	0.034	0.034
Red sweet pepper powder	0.049	0.049	0.049
Garlic powder	0.025	0.025	0.025
Polyphosphate	0.350	0.350	0.350
Red wine	2.006	2.006	2.006
Water	3.478	3.478	3.478

PBF—porkbackfat, GM_OG—glycerol monostearate oleogel, CW_OG—candelilla wax oleogel.

## Data Availability

The original contributions presented in the study are included in the article.
